# The Role of Dietary Anthocyanins for Managing Diabetes Mellitus-Associated Complications

**DOI:** 10.2174/0115733998322754240802063730

**Published:** 2024-08-12

**Authors:** Priya S. Mistry, Mehul R. Chorawala, Bhagavathi Sundaram Sivamaruthi, Bhupendra G. Prajapati, Akash Kumar, Chaiyavat Chaiyasut

**Affiliations:** 1 Department of Pharmacology and Pharmacy Practice, L. M. College of Pharmacy, Opp. Gujarat University, Navrangpura, Ahmedabad 380009, Gujarat, India;; 2 Office of Research Administration, Chiang Mai University, Chiang Mai 50200, Thailand;; 3 Innovation Center for Holistic Health, Nutraceuticals, and Cosmeceuticals, Faculty of Pharmacy, Chiang Mai University, Chiang Mai 50200, Thailand;; 4 Department of Pharmaceutics and Pharmaceutical Technology, Shree S. K. Patel College of Pharmaceutical Education & Research, Ganpat University, Mehsana, Gujarat, India;; 5 MM Institute of Hotel Management, Maharishi Markandeshwar (Deemed to be University), Mullana 133207, India;; 6 Department of Food Technology, SRM University, Delhi-NCR, Sonepat 131029, India

**Keywords:** Anthocyanins, diabetes mellitus, antioxidant, inflammation, insulin sensitivity, oxidative stress

## Abstract

Diabetes mellitus (DM) is an intricate metabolic disorder marked by persistent hyperglycemia, arising from disruptions in glucose metabolism, with two main forms, type 1 and type 2, involving distinct etiologies affecting β-cell destruction or insulin levels and sensitivity. The islets of Langerhans, particularly β-cells and α-cells, play a pivotal role in glucose regulation, and both DM types lead to severe complications, including retinopathy, nephropathy, and neuropathy. Plant-derived anthocyanins, rich in anti-inflammatory and antioxidant properties, show promise in mitigating DM-related complications, providing a potential avenue for prevention and treatment. Medicinal herbs, fruits, and vegetables, abundant in bioactive compounds like phenolics, offer diverse benefits, including glucose regulation and anti-inflammatory, antioxidant, anticancer, anti-mutagenic, and neuroprotective properties. Anthocyanins, a subgroup of polyphenols, exhibit diverse isoforms and biosynthesis involving glycosylation, making them potential natural replacements for synthetic food colorants. Clinical trials demonstrate the efficacy and safety of anthocyanins in controlling glucose, reducing oxidative stress, and enhancing insulin sensitivity in diabetic patients, emphasizing their therapeutic potential. Preclinical studies revealed their multifaceted mechanisms, positioning anthocyanins as promising bioactive compounds for managing diabetes and its associated complications, including retinopathy, nephropathy, and neuropathy.

## 1 INTRODUCTION

Diabetes mellitus (DM) represents a global health challenge with an escalating prevalence and profound implications for public health [1]. The global prevalence of diabetes will be 783.2 million in 2045, with 11.9, 21.1 and 12.2% increases in low, middle, and high-income countries, respectively [2]. Diabetes instigates a deleterious cycle marked by persistent hyperglycemia, setting in motion a cascade of metabolic disruptions [3]. This chronic elevation of blood glucose levels fosters an impaired metabolic state, triggering events that culminate in non-enzymatic glycation of proteins and lipids [4]. This complex process unfolds as elevated glucose levels react with cellular proteins and lipids, forming advanced glycation end-products (AGEs) [5]. Small blood vessels in these crucial tissues bear the brunt of this glycation-induced damage and thereby significantly increase the risk of various complications associated with diabetes, including cardiovascular diseases, nephropathy, neuropathy, and retinopathy [6]. This intricate interplay underscores the urgency of glycemic control, as elevated glucose levels perpetuate hyperglycemia and expedite the formation of AGEs [7]. In essence, the relentless cycle of hyperglycemia and subsequent glycation forms the crux of classic diabetic complications [8]. Understanding this complex interplay not only elucidates the underlying pathophysiology but also emphasizes the pivotal role of stringent glycemic control in averting the cascade of detrimental outcomes in diabetes.

In the multifaceted landscape of diabetes management, a two-pronged approach involving non-pharmacological and pharmacological interventions is paramount [9-11]. Non-pharmacological strategies, encompassing dietary modifications and increased physical activity, lay the foundation for optimal glycemic control [12]. Concurrently, pharmacological treatments, including widely used anti-diabetic drugs like sulfonylureas, biguanides, α-glucosidase inhibitors, thiazolidinediones, insulin, and many novel agents, provide crucial support in regulating blood glucose levels [13]. Despite advances in therapeutic interventions, the management of diabetes and its associated complications remains a complex and evolving field. While conventional anti-diabetic drugs play a pivotal role in managing blood sugar levels and, thereby, complications, they are not devoid of side effects [14]. This highlights the growing need for novel agents sourced from natural reservoirs that prove effective in glycemic control and ensure safety in long-term usage. Among these, anthocyanins have emerged as promising candidates due to their diverse biological activities, including anti-inflammatory and antioxidant properties, with their multifaceted benefits and origins in plant-based sources as a promising avenue in this pursuit, offering a potential synergy of efficacy and safety in the ongoing quest for effective and side-effect-minimized diabetes management strategies [15].

Various nutritional factors, such as diets, fermented foods, herbs, and supplements, have positively affected the consequences of diabetes [16-22].

Anthocyanins are water-soluble pigments widely distributed in fruits, vegetables, and other plant-based foods, imparting vibrant colors, such as red, purple, and blue [23]. Recent studies on anthocyanins, naturally occurring pigments in plants, have unveiled their promising effects on various facets of DM [24-28]. Anthocyanins significantly affect glucose metabolism, insulin resistance, and lipid metabolism and exhibit potent anti-inflammatory and antioxidant properties [25]. These attributes position anthocyanins as potential contributors to the prevention and treatment of DM. Notably, research suggests that anthocyanins may extend their positive influence beyond glycemic control, improving organ function and acting as a preventive measure against diabetes-associated complications [29].

The rationale for the present review article stems from the growing body of evidence suggesting that dietary anthocyanins may significantly mitigate diabetes and associated complications. While preclinical studies have demonstrated encouraging results, translating these findings to clinical applications and understanding the underlying mechanisms require a comprehensive examination. This review aims to summarize existing knowledge, providing insights into the potential mechanisms by which anthocyanins exert their effects, summarizing evidence from preclinical and clinical studies, and exploring their impact on specific complications associated with diabetes. The scope of this exploration encompasses a multidimensional analysis, starting with an investigation into the bioavailability and metabolism of anthocyanins. It will then elucidate the mechanisms through which these natural compounds exert their effects, focusing on anti-inflammatory, antioxidant, and metabolic pathways pertinent to diabetes. The review will critically assess preclinical studies, offering insights into the impact of anthocyanins on diabetes-related complications in animal models, with attention to key findings and potential limitations.

Furthermore, a synthesis of clinical trial outcomes will be undertaken, shedding light on the efficacy of anthocyanin consumption in human subjects while considering methodological variations and diverse outcomes. Specific emphasis will be placed on the influence of anthocyanins on distinct complications associated with diabetes, such as cardiovascular diseases, nephropathy, neuropathy, and retinopathy. Additionally, the review aims to investigate the potential synergistic effects and safety considerations of combining anthocyanin-rich diets with conventional anti-diabetic medications, presenting an integrated perspective on therapeutic approaches. The overarching objective is to provide a nuanced understanding of the role of dietary anthocyanins in managing diabetes mellitus-associated complications. By identifying gaps in existing knowledge and proposing future directions for research, this review aspires to contribute valuable insights for researchers, clinicians, and policymakers keen on exploring holistic and nature-derived approaches to diabetes care.

Scientific literature was collected from the databases (PubMed, Scopus, and Google Scholar) using the keywords “Anthocyanins,” “Diabetes,” “Anthocyanins and diabetes,” and “Diabetes mellitus.” The studies published in English with complete access have been selected without time limits.

## 2 ANTHOCYANINS: NATURE'S COLORFUL COMPOUNDS

Growing concerns regarding the side effects associated with pharmaceutical drugs have spurred a heightened interest in exploring plant-based alternatives [30]. Regularly consuming medicinal herbs, fruits, and vegetables is widely acknowledged for its positive impact on human health [31]. Abundant in non-nutrient bioactive compounds, particularly phenolics, these plant-derived foods offer a spectrum of benefits, including regulating glucose levels and enhancing anti-inflammatory, antioxidant, anticancer, anti-mutagenic, and neuroprotective properties [32]. Polyphenols are sometimes vibrant pigments and a captivating class of flavonoids found abundantly in plants [33]. Anthocyanins hold more than just aesthetic significance because they defend against environmental stressors and contribute to the visual allure of fruits, vegetables, and flowers.

### 2.1 Definition and Classification

Anthocyanins are a group of water-soluble pigments found in natural plants responsible for vivid colors such as red, pink, orange, violet, and blue observed in vegetables, fruits, leaves, and flowers [34]. Some of these edible pigmented flowers have proven medicinal use [35]. Structurally, anthocyanins consist of a flavonoid core with an anthocyanidin molecule linked to one or more sugar moieties. The diversity in anthocyanin structures arises from variations in the anthocyanidin aglycone, the type and number of sugar moieties, and the degree of glycosylation [35]. Beneath their colorful facade lies a rich tapestry of bioactive properties, with emerging research highlighting their potential health benefits, particularly in diabetes mellitus and associated complications [36].

Anthocyanins can be classified based on several criteria, including the aglycone type, the glycosylation degree, and the acylation pattern [35]. The primary classification is based on anthocyanidin aglycone, where major types include cyanidin, delphinidin, pelargonidin, petunidin, peonidin, and malvidin [37]. These different aglycones isoforms contribute distinct colors to the anthocyanins they form, influencing the overall coloration of the plant tissues and giving rise to the rich visual palette observed in nature [34]. Another classification aspect considers the glycosylation pattern, with anthocyanins often found in glycosidic forms [38]. Monoglycosides, diglycosides, and triglycerides refer to the number of sugar molecules attached to the anthocyanidin core [34]. The specific sugars and their arrangement further contribute to the diversity within this classification.

Additionally, acylation, the attachment of organic acid groups, contributes to anthocyanin variability. Acylated anthocyanins often exhibit altered color stability and may have different physiological roles in plants [39]. Understanding the classification of anthocyanins is crucial for unraveling their diverse functions in plants and harnessing their potential health benefits, as different anthocyanins may have distinct bioactivities and stabilities [40]. Table **1** details various anthocyanidin isoforms and their respective pigment colors. Anthocyanidins, the aglycones of anthocyanins, contribute distinct hues to plant tissues, enriching the visual spectrum with shades ranging from reddish-purple and magenta to blue-reddish, red-orange, and dark red or purple [41]. Understanding the association between anthocyanidin isoforms and pigment colors provides insights into the chromatic diversity exhibited by these natural compounds.

### 2.2 Structure and Synthesis of Anthocyanins

**Fig. (1):**
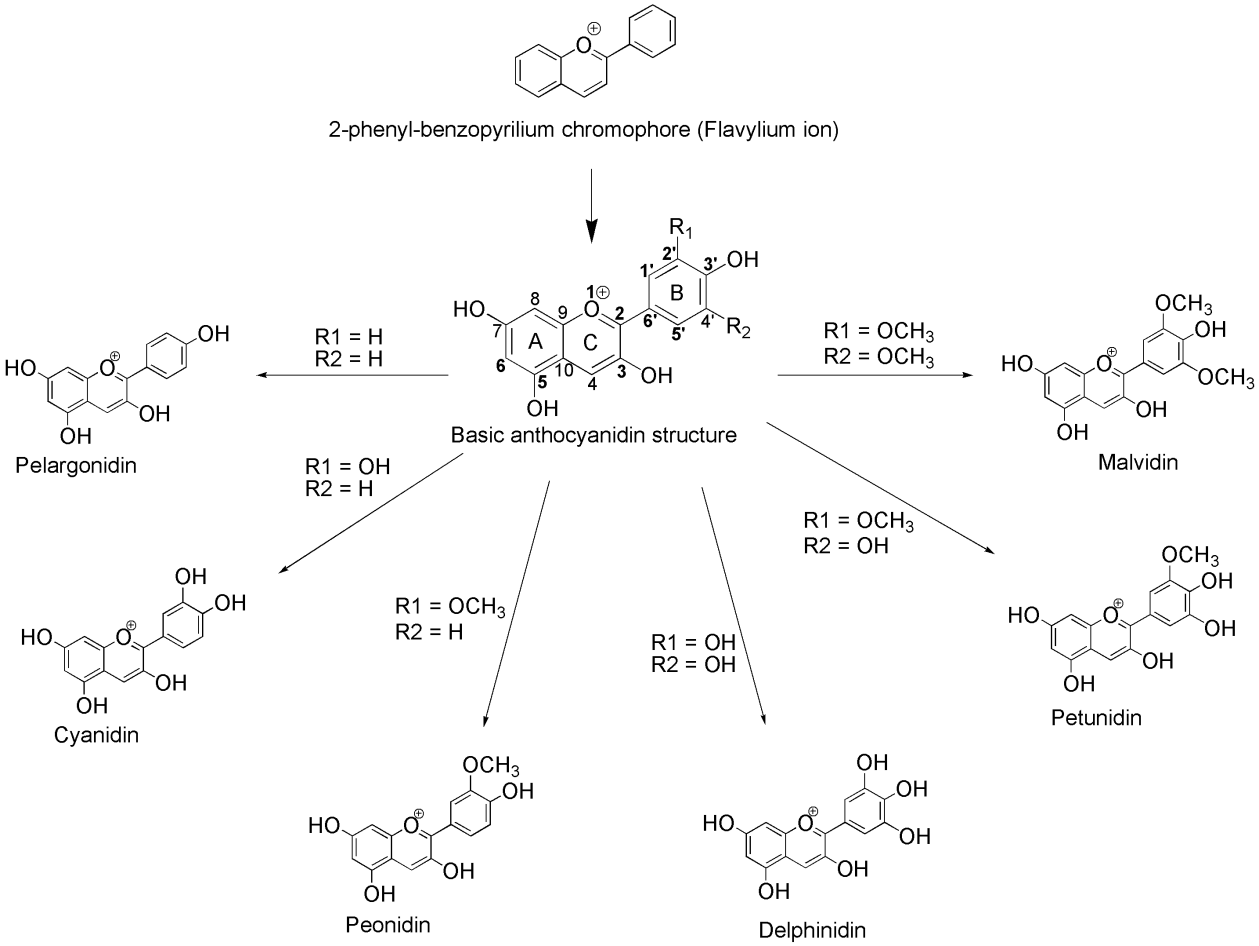
Biosynthesis of anthocyanins: Glycosylation and structural diversity.

Anthocyanins, classified among the extensive group of flavonoids, represent secondary metabolites in plants. Within the vast repository of over 5000 flavonoids, a subset of 20 natural anthocyanins has been identified to date [42]. The foundational structure of flavonoids comprises two aromatic rings intricately connected by a C3 pyran ring- a heterocyclic, six-membered non-aromatic ring composed of five carbon atoms and one oxygen atom, featuring two double bonds [43]. Anthocyanins and anthocyanidins emerge from the fundamental structure of the 2-phenyl-benzopyrilium chromophore (flavylium ion) by substituting the flavylium cation [34]. Anthocyanidins, exemplified by the cationic structure 3, 5, 7-trihydroxyl-2-phenylbenzo-pyran (Fig. **1**), delineate a complex arrangement [44]. This structure manifests an aromatic ring A intricately linked to an oxygen-containing heterocyclic ring C [45].

Furthermore, ring C establishes a carbon-carbon bond with a third aromatic ring B. Such structural intricacies underscore the chemical richness of anthocyanins, laying the groundwork for a detailed comprehension of their biosynthesis, functional properties, and diverse roles in plant physiology [46]. Anthocyanidins undergo glycosylation with mono-, di-, or trisaccharides at distinct sites, resulting in the structural diversity observed in anthocyanins. The number and arrangement of linked sugar moieties (glycosylation) and the type and quantity of attached acids (acylation) contribute to the multifaceted nature of anthocyanin derivatives.

The intricate biosynthesis of anthocyanins involves the glycosylation of anthocyanidins, where hydroxyl groups (-OH) on the anthocyanidin scaffold undergo partial or total substitution by sugar moieties [34]. Typically, anthocyanins exist as heterosides, and glycosylation occurs with varying degrees, resulting in mono-, di-, and trisaccharides. Sugar moieties attach to anthocyanidins at inconsistent sites, yielding 3-aminoglycosides or 3,5-diglycosides [47]. The prevailing monosaccharide is glucose, accompanied by rhamnose, galactose, arabinose, xylose, and fructose. Thus, anthocyanins emerge as glycosylated derivatives of flavylium salts, either polyhydroxy or polymethoxy [48]. The remarkable diversity observed among anthocyanins results from the number and arrangement of linked sugar moieties (glycosylation) and the presence, type, and quantity of aliphatic or aromatic acids (acylation) attached to the anthocyanidin core [35]. Monoglycosides predominantly exhibit a single glycosyl attachment, typically at the C3 site [49]. Diglycosides, on the other hand, display two glycosyls consecutively at the C3 or C3 and C5 sites, occasionally extending to the C3 and C7 sites [50]. Triglycosides commonly involve two glycosyls attached sequentially at the C3 site, with the third at the C5 or C7 site [50]. Notably, the simultaneous attachment of all three glycosyls at the C3 site results in a branched or linear sugar chain [51]. However, the attachment of three glycosyls at the C7 site is a rarity in anthocyanin structures. This glycosylation intricacy contributes significantly to anthocyanins' structural diversity and functional characteristics, highlighting their dynamic nature within plant metabolomes [52].

### 2.3 Dietary Sources of Anthocyanins

Beyond their aesthetic contributions, anthocyanins have garnered attention for their potential health benefits in preventing various diseases. Anthocyanins are abundantly present in diverse foods, enriching the color palette of fruits, vegetables, beans, cereals, and nuts. Notable sources include berries (such as strawberries, blueberries, and raspberries), cherries, grapes, apples, and plums, as well as dark-colored vegetables like red radish, red onion, eggplants, red cabbage, beans, and purple sweet potatoes. Grains like black rice, purple maize, and red sorghum also contribute to anthocyanin content in the diet [32, 53].

Among the more than 600 identified anthocyanins, C3G is the most abundant in fruits and vegetables [34]. Anthocyanin is formed under acidic conditions, which explains its prevalence in acidic fruits like berries. Other glycosides, including malvidin, peonidin, and petunidin, are also present in various fruits and vegetables, contributing to their distinct colors [54, 55]. Notably, petunidin glycosides are commonly found in purple-colored vegetables, although their health benefits are less widely recognized [34]. Malvidin, on the other hand, boasts high antioxidant properties with remarkable bioavailability [56]. Anthocyanin-rich foods are easily identifiable due to their specific pigment properties, showcasing a spectrum of red, blue, and purple hues. Notable exceptions include red tomatoes, which derive their color from lycopene, and beets, characterized by betalain pigments [57]. In brief, the intricate world of anthocyanins in food extends beyond their role as natural colorants. From their diverse dietary sources to the specific anthocyanins responsible for their vibrant hues, understanding the nuances of these compounds contributes to both the aesthetic appeal of our meals and the potential health benefits associated with their consumption. Table **2** provides a comprehensive overview of anthocyanin composition in diverse fruits.

### 2.4 Bioavailability and Metabolism

Anthocyanins, vibrant pigments abundant in various fruits, vegetables, and grains, have garnered significant attention for their potential health benefits [34]. It is crucial to unravel the intricate processes of bioavailability and metabolism that anthocyanins undergo within the human body. Several factors influence the bioavailability of anthocyanins, ranging from the chemical structure of the compounds to interactions with other dietary components [58]. Food components such as fat and alcohol, as well as cooking processes like microwaving, steam-blanching, juice-making, and fermentation, affect the bioavailability of anthocyanins [45, 58-60].

Upon ingestion, anthocyanins undergo digestion in the gastrointestinal tract. The stomach's acidic environment plays a vital role in the stability and transformation of anthocyanins [61]. The glycosidic bonds that attach sugar moieties to the anthocyanidin core are susceptible to hydrolysis under acidic conditions. This process leads to the release of anthocyanidins, the aglycones, which are more readily absorbed [40]. Following gastric digestion, anthocyanins move to the small intestine, where absorption occurs. Most anthocyanins are absorbed in the small intestine through passive diffusion, facilitated by their lipophilic nature. However, glycosylation can hinder this process, particularly with larger sugar moieties. Once absorbed, anthocyanins enter the portal circulation and reach the liver, where further metabolism occurs [62]. Conjugation with glucuronic acid or sulphate takes place, contributing to the water solubility of anthocyanins and facilitating their excretion [34]. The bioavailability of anthocyanins is influenced by the food matrix in which they are consumed. Co-ingestion with certain macronutrients, such as lipids, can enhance absorption. Additionally, dietary fiber may influence the transit time in the gastrointestinal tract, affecting the exposure of anthocyanins to porous surfaces [63].

Anthocyanin metabolism involves a series of enzymatic and non-enzymatic reactions that transform these compounds into various metabolites. The liver is a central site for anthocyanin metabolism, where phase II enzymes play a crucial role in conjugation reactions [35]. One of the primary metabolic pathways involves the glucuronidation of anthocyanins. Glucuronidation is a phase II conjugation reaction where glucuronic acid is attached to anthocyanins' aglycone or sugar moieties [64]. This process enhances water solubility and facilitates excretion. Sulfation is another important metabolic pathway. In this process, sulphate groups are added to anthocyanins, increasing their water solubility. Both glucuronidation and sulfation contribute to forming more polar metabolites, which are readily excreted through urine. In addition to enzymatic conjugation, anthocyanins can undergo non-enzymatic reactions [40]. Methylation, for example, involves adding a methyl group to the anthocyanidin core, leading to the formation of methylated anthocyanins. These methylated forms also contribute to the overall metabolic profile of anthocyanins. The metabolites formed through these processes are often more stable than the parent anthocyanins and may exhibit different bioactivities [65]. The intricate network of enzymatic and non-enzymatic transformations highlights the dynamic nature of anthocyanin metabolism.

The final step in the journey of anthocyanins within the human body is excretion. The water-soluble metabolites, formed through conjugation reactions in the liver, are primarily excreted through urine. This renal excretion is crucial to eliminating anthocyanin-derived compounds from the body. Some studies suggest that a small fraction of anthocyanins may undergo enterohepatic circulation, and these compounds may be reabsorbed in the intestines after excretion and re-enter the circulation. The enterohepatic circulation could extend the duration of anthocyanin in the body and influence its overall bioactivity [66].

From the initial digestion and absorption in the gastrointestinal tract to the phase II metabolism in the liver and eventual excretion, anthocyanins undergo a complex journey within the human body. Understanding these processes is fundamental for harnessing the potential health benefits of consuming anthocyanin-rich foods and supplements. As research in this field progresses, insights into the bioavailability and metabolism of anthocyanins continue to deepen, offering a nuanced understanding of their impact on human health. The intricate interplay between the chemical structure of anthocyanins, dietary factors, and individual variations underscores the complexity of their journey within the body [61, 67, 68].

## 3 DIABETES MELLITUS

DM, a most common form of endocrinal-metabolic disorder, is associated with impaired glucose metabolism, leading to persistent elevation in blood glucose levels. Based on pathophysiology, diabetes is categorized into two major forms: type 1 diabetes (T1DM) and type 2 diabetes (T2DM) [69]. Other categories include mature-onset diabetes of the young (MODY), [70] gestational neonatal, [71] and secondary causes due to several conditions like endocrinopathies or drug-induced such as steroids [72]. The islets of Langerhans have two main subclasses of cells: β-Cells that produce insulin and α-Cells that secrete glucagon [73]. The action of both cells depends on and is regulated by the glucose environment [74]. The prime difference between the two types of DM lies in their etiology. In T1DM, the β-cells are destroyed, leading to the complete absence of insulin or extremely low insulin levels [75]. T2DM is characterized by insulin insensitivity due to insulin resistance [75]. Insulin sensitivity is typically defined as the capacity of insulin to decrease plasma glucose levels, achieved by inhibiting hepatic glucose production and promoting glucose uptake in skeletal muscle and adipose tissue (Wilcox, 2005). Insulin resistance is fundamentally a state of diminished insulin sensitivity. Insulin resistance denotes a compromised biological responsiveness to insulin [76]. Both forms of diabetes ultimately result in hyperglycemia [75]. Consequently, a detrimental cycle ensues, wherein hyperglycemia contributes to a compromised metabolic state [77].

The maintenance of glucose homeostasis ensures normal body function. Peptides secreted by the pancreas, such as insulin and glucagon, play a vital role in maintaining this glucose homeostasis, and an imbalance in it is related to the onset of hyperglycemia and DM [78]. In normal glucose homeostasis, digestive enzymes like α-amylase and α-glucosidase hydrolyze carbohydrates into monosaccharides, which are then transported across the small intestine, resulting in an increased blood glucose level and glucagon-like peptide 1 (GLP-1). In response to this increase in glucose and GFLP-1, pancreatic β-cells rapidly secrete insulin, which regulates various tissues like liver muscle and adipose tissue to begin the glucose uptake [79]. Additionally, insulin guides the liver to initiate glycogenesis and inhibition of gluconeogenesis.

In contrast, pancreatic α-cells secrete glucagon that increases blood glucose by increasing glucose production by glycogenolysis and gluconeogenesis [80]. However, prolonged increases in insulin levels may lead to a loss of response from tissues for glucose uptake, which is referred to as insulin resistance, typically observed in T2DM [81]. Thus, insufficient or absence of insulin secretion leads to the development of DM due to high glucose levels. In understanding the intricate relationship of pancreatic peptides regulating glucose homeostasis, refer to Fig. (**2**) for a visual representation of the dynamic interplay between insulin, glucagon, and digestive enzymes.

DM is associated with several acute (Diabetic ketoacidosis and coma, hyperglycemia) and chronic (Macroangiopathy, diabetic retinopathy, nephropathy, neuropathy, diabetic foot, heat diseases) complications [82]. Timely diagnosis plays a pivotal role in attaining predefined objectives in managing DM. A fundamental component of DM management involves lifestyle modifications encompassing reducing sedentary behaviour, increased engagement in physical activities, and adherence to healthy dietary practices. The exclusive pharmacological intervention for T1DM is insulin therapy [83]. In contrast, the initial approach for T2DM incorporates lifestyle adjustments and monotherapy utilizing oral hypoglycemic agents (OHAs), typically featuring metformin from the biguanide class [84, 85]. Additional OHAs include GLP-1 receptor agonists (GLP-1 RAs) such as albiglutide, dulaglutide, exenatide, liraglutide, and SGLT2 inhibitors (including dapagliflozin, canagliflozin, and empagliflozin), DPP-4 (dipeptidyl peptidase 4) inhibitors (Alogliptin, linagliptin, and sitagliptin), sulfonylureas such as glimepiride, glipizide, and glyburide, and TZDs (Pioglitazone, Rosiglitazone) [86, 87]. Recent advancements in diabetes prevention and treatment encompass nanotechnology utilizing nanoparticles (<100 nm) for non-invasive insulin delivery, the development of more potent vaccines involving cell-based and gene-based therapies for T1DM [88, 89], and medical nutrition therapy (MNT), a nutrition-focused treatment administered by a registered dietitian nutritionist to maintain normal blood glucose levels. Furthermore, gene therapy targeting various entities like GLUTs, SGLTs, GLP-1, *etc*., has emerged as a recognized approach for DM management [90, 91].

## 4 ANTHOCYANINS AND THEIR MECHANISMS OF ACTION

The two prime causes of diabetic complications in various tissues and organs are oxidative stress and inflammation [3, 92]. Findings have shown that the anti-hyperglycaemic effect of Anthocyanins is due to their action on cellular and molecular targets like intestinal glucose transport, carbohydrate hydrolyzing enzymes, hepatic glucose metabolism, gut hormones, islet function, and insulin resistance [93]. Anthocyanins have also been established as useful in ameliorating diabetic complications [29]. Anthocyanins are only effective when there is a pre-existing metabolic disorder. Thus, its effect on lowering blood glucose in non-diabetic humans would be insignificant.

The pathogenesis of T1DM is autoimmunity, which releases inflammatory cytokines and together cause destruction or damage to islet cells in the pancreas [94]. It results in disease progression. The main enzyme responsible for triggering inflammation is cyclooxygenase-2 (COX-2), and for oxidative stress is inducible nitric oxide (NO) synthase (iNOS) that synthesizes large amounts of NO [95].

Anthocyanins have been known to block mRNA expression of COX-2 and iNOS and improve the expression of antioxidant enzymes such as superoxide dismutases (SOD), glutathione (GSH), and catalase (CAT) [96]. Thus improving oxidative stress and inflammation. T2DM is a non-insulin-dependent type of DM and is associated with insulin resistance, decreased insulin sensitivity, and lipid metabolism disorder, which lead to hyperglycemia, oxidative stress, and inflammation. Thus, targets to prevent further progression of T2Dm include improving insulin resistance, blood glucose levels, lipid metabolism, and oxidative stress and inflammation [4].

Anthocyanins are known to improve and prevent further progression of T2DM. The known mechanisms of anthocyanins include a) inhibition of carbohydrate metabolizing enzymes and intestinal glucose transport, b) inhibition of dipeptidyl peptidase IV (DPP-4), c) protection of β-cells, d) improvement of insulin resistance, e) improvement of blood glucose levels, f) improving lipid metabolism g) reducing oxidative stress and h) reducing inflammation [93, 96-98]. Refer to Fig. (**3**) for an illustrative overview of the multifaceted mechanisms by which anthocyanins benefit diabetes, encompassing anti-inflammatory, antioxidant, and metabolic regulation pathways.

### 4.1 Anti-inflammatory Effects

The anti-inflammatory mechanism of anthocyanins in diabetes involves intricate molecular pathways and interactions that modulate oxidative stress, immune response, and inflammatory signaling. Firstly, anthocyanins exert their anti-inflammatory effects through potent antioxidant activity [99]. In diabetes, there is an imbalance between reactive oxygen species (ROS) production and the body's ability to neutralize them [100]. Anthocyanins act as scavengers of ROS, mitigating oxidative stress and reducing the damage inflicted on cellular structures. This antioxidative capacity is essential for suppressing inflammation, as oxidative stress is a crucial contributor to the chronic inflammatory state observed in diabetes [101]. Secondly, anthocyanins play a pivotal role in modulating inflammatory signaling pathways. The nuclear factor-kappa B (NF-κB) pathway, a central regulator of inflammation, is often dysregulated in diabetes [102]. Anthocyanins have been shown to inhibit NF-κB activation, downregulating proinflammatory cytokine and adhesion molecules' expression [103]. This interference in NF-κB signaling is a key mechanism by which anthocyanins exert their anti-inflammatory influence in the diabetic context.

Furthermore, anthocyanins directly affect immune cells, influencing the immune response associated with diabetes-related inflammation [104]. These compounds can modulate the activity of immune cells, including macrophages and T lymphocytes, thereby regulating the production of inflammatory mediators. By fine-tuning the immune response, anthocyanins attenuate chronic inflammation in diabetes. In addition, endothelial dysfunction, a common complication in diabetes, is mitigated by anthocyanins through improvement of nitric oxide bioavailability [105]. Enhanced nitric oxide production leads to vasodilation and improved endothelial function, reducing inflammation in the vascular system. This effect is crucial in preventing complications such as cardiovascular diseases that are exacerbated by inflammation in diabetes [106].

Moreover, anthocyanins may indirectly contribute to anti-inflammatory effects by improving insulin sensitivity. Insulin resistance is closely associated with inflammation in diabetes, and anthocyanins have been implicated in enhancing insulin action [107]. By promoting insulin sensitivity, anthocyanins contribute to the regulation of glucose metabolism and the subsequent reduction of inflammation associated with insulin resistance.

### 4.2 Antioxidant Properties

The primary mechanism of anthocyanins to decrease oxidative stress involves their ability to scavenge ROS, thereby preventing oxidative damage to cellular components. Their structure allows them to efficiently donate electrons and neutralize free radicals, such as superoxide anions and hydroxyl radicals, which are often elevated in diabetes due to hyperglycemia-induced oxidative stress [108]. Moreover, anthocyanins can chelate metal ions, particularly transition metals like iron and copper, which are known to catalyze the generation of ROS through Fenton reactions [109]. By sequestering these metal ions, anthocyanins interrupt the pro-oxidative cascade, attenuating the formation of highly reactive hydroxyl radicals [110]. The metal-chelating property directly mitigates oxidative stress and disrupts the positive feedback loop that sustains ROS production in diabetic conditions. Another facet of the antioxidant mechanism involves the modulation of cellular signaling pathways. Anthocyanins have been shown to influence the nuclear factor erythroid 2-related factor 2 (Nrf2) pathways, a key regulator of the antioxidant response [111]. Under oxidative stress conditions, Nrf2 dissociates from its inhibitor, Kelch-like ECH-associated protein 1 (Keap1), translocating into the nucleus, orchestrating the transcription of antioxidant genes [112]. Anthocyanins enhance this process by promoting the dissociation of Nrf2 from Keap1, thereby upregulating the expression of antioxidant enzymes such as heme oxygenase-1 (HO-1) and SOD [113]. This augmentation of the endogenous antioxidant defense system fortifies the cell's ability to combat oxidative stress associated with diabetes [112].

Furthermore, anthocyanins exhibit anti-inflammatory effects, contributing to their antioxidant activity in diabetes. Chronic inflammation is intricately linked to oxidative stress in diabetes mellitus. Anthocyanins interfere with inflammatory signaling pathways, such as nuclear factor-kappa B (NF-κB), by inhibiting the activation of proinflammatory cytokines and adhesion molecules [99, 104]. The anti-inflammatory action not only mitigates the inflammatory component of diabetes but also indirectly reduces the oxidative stress associated with chronic inflammation. The mitochondria, central to energy metabolism, are pivotal in diabetes-associated oxidative stress. Anthocyanins have been demonstrated to protect mitochondria from dysfunction, maintaining their structural integrity and functional efficiency. By preserving mitochondrial function, anthocyanins reduce the leakage of electrons in the respiratory chain, thereby diminishing the production of ROS at the mitochondrial level. Additionally, anthocyanins may enhance mitochondrial biogenesis, promoting the generation of new and healthy mitochondria [114]. This multifaceted approach underscores the potential of anthocyanins to address diabetes-induced oxidative stress at its source. Anthocyanins have enhanced insulin sensitivity, which is critical to glycaemic control in diabetes [25]. Improved insulin sensitivity reduces the burden of hyperglycemia, indirectly contributing to the attenuation of oxidative stress linked to elevated glucose levels. While the precise molecular pathways involved in the insulin-sensitizing effects of anthocyanins are not fully elucidated, evidence suggests their role in modulating insulin signaling cascades and glucose transporter expression.

### 4.3 Impact on Insulin Sensitivity and Glucose Metabolism

Hyperinsulinemia causes reduced insulin sensitivity and a decrease in glucose uptake. Insulin resistance in the liver and various peripheral tissues and insufficient insulin secretion by β-cells play a major role in the development of DM [115]. Diabetes thereby leads to oxidative stress and de novo lipid synthesis, which causes liver disorder and complications [116]. Improving insulin resistance in peripheral tissue and liver would increase insulin sensitivity and thus is another target for preventing and treating hyperglycemia. Anthocyanins present in berries were shown to decrease oxidative stress and regulate glucose metabolism, thus regulating blood glucose levels [117]. Cellular hepatic glucose and lipid metabolism are checked by AMP-activated protein kinase (AMPK), whereas the glucose uptake, repression of gluconeogenesis in hepatocytes, and glycogen synthesis are maintained by protein kinase B (Akt) [118]. Anthocyanins from various sources, like berry extracts, can modify the expression of glucose transporter protein type-4 (GLUT4), stimulate the AMPK pathway, and decrease oxidative stress, eventually improving insulin sensitivity and positively modulating glycemic control [118]. PI3K/Akt pathway is an insulin signaling pathway. This signaling causes increased glycogen synthesis and a decrease in gluconeogenesis [119]. Proinflammatory cytokines activate the Kappa B kinase (IKK)/ NF-kB pathway inhibitor, further suppressing the PI3K/Akt pathway to improve insulin resistance [120]. Another mechanism that leads to insulin resistance is the phosphorylation of insulin receptor substrate 1 (IRS-1) serine residues [121]. Anthocyanins inhibit the activation of Jun NH2-terminal kinase (JNK) and NF-kB, leading to decreased phosphorylation of IRS-1 serine residues [122]. Apart from the abovementioned mechanisms, anthocyanins induce adiponectin, an insulin sensitizer [123]. Therefore, this mechanism improves insulin resistance. Anthocyanins found in mulberry commonly include C3G, cyanidin 3-rutinoside, and pelargonidin 3-glucoside, which are known to increase glucose uptake and consumption [124]. Treatment with anthocyanins extract showed a decrease in phosphoenolpyruvate carboxykinase and glucose-6-phosphatase due to inhibition of peroxisome proliferator-activated receptor-gamma (PPARγ), forkhead box protein O1, and coactivator 1α in HepG2 cells [125, 126]. Mulberry extract also inhibits gluconeogenesis and activates the PI3K/Akt pathway, thus regulating glucose metabolism [127].

### 4.4 Other Potential Mechanisms

#### 4.4.1 Inhibition of Carbohydrate Metabolizing Enzymes and Intestinal Glucose Transport

Carbohydrate digestion takes place with the help of two carbohydrate-metabolizing enzymes: α-amylase and α-glucosidase [128]. The macromolecules are catalyzed to oligosaccharides by α-amylase, which are further catalyzed to monosaccharides by α-glucosidase, and these monosaccharides undergo absorption and transport to blood [129]. The increase in bioavailability leads to increased blood glucose levels. The transporter involved in the absorption of monosaccharides includes sodium-glucose transporter 1 (SGLT1) and glucose transporter 2 (GLUT2) [130]. Therefore, inhibition of α-amylase, α-glucosidase, SGLT1, and GLUT2 will reduce post-prandial glucose and the gut's glucose level [131], relieving hyperglycemia and averting complications from diabetes. Anthocyanins from food sources like rich berries effectively inhibit α-amylase and α-glucosidase activities, interacting with SGLT1 and GLUT2, thereby decreasing intestinal glucose absorption [132]. Petunidin 3-glucoside, delphinidin 3-glucoside, C3G, and pelargonidin 3-glucoside have inhibitory action against these enzymes [133]. Of the abovementioned Anthocyanins, delphinidin 3-glucoside and petunidin 3-glucoside were more active than acarbose in inhibiting α-glucosidase [134]. The order of individual anthocyanins inhibiting the carbohydrate metabolizing enzyme is C3G > cyanidin 3-rutinoside > C3G > peonidin 3-glucoside [135]. In an *in vitro* study, the most potent inhibitor of α-amylase and α-glucosidase was pelargonidin 3-rutinoside [136].

#### 4.4.2 Inhibition of DPP4 and Regulation Of Insulin Secretion

Incretins such as GLP-1 are released in the blood after food ingestion to improve glucose-induced insulin secretion and inhibit glucagon secretion [137]. This increase in insulin levels and decrease in glucagon levels lead to increased glycogenesis and reduced gluconeogenesis, decreasing post-prandial glycemia [138]. DPP-4 converts GLP-1 to its inactive form, decreasing its efficacy GLP-1 to increase insulin and decrease glucagon secretion [139]. Two main characteristics of T2DM are inadequate insulin secretion by β-cells and insulin resistance [140]. Thus, the protection of β-cells is also vital to maintain its function. Various studies have shown the effects of anthocyanin in inhibiting DPP-4 [141]. Extracts of anthocyanin from *Lonicera caerulea* and blueberry-blackberry beverages were shown to inhibit DPP-4 [142]. The major anthocyanins present in blueberry and blackberry are malvidin 3-galactoside and delphinidin 3-arabinoside, respectively, which proved to be potent inhibitors of DPP-4 [143]. Another *in vitro* study investigated the inhibitory action of aronia anthocyanins, which was in the order of cyanidin 3,5-diglucoside > C3G > cyanidin [144]. However, there is a gap between understanding the structure-activity relationship and the inhibitory mechanism of DPP-4.

#### 4.4.3 Β-Cell Cytoprotection

Pancreatic β-cells play a crucial role in sensing and releasing the appropriate amount of insulin in response to glucose stimuli. Due to their low levels of antioxidant enzymes like CAT, SOD, and glutathione peroxidase, β-cells are susceptible to oxidative stress induced by hyperglycemia [145]. Research indicates that dietary antioxidants can protect pancreatic β-cells from glucose-induced oxidative stress. Numerous *in vitro* and *in vivo* studies link the reduction of oxidative stress through anthocyanins to increased insulin production in pancreatic β-cells of individuals with type-2 diabetes [146-148].

In a comparative study evaluating the impact of various anthocyanins and anthocyanidins on insulin secretion from rodent pancreatic β-cells (INS-1 832/13), C3G and delphinidin-3-glucoside proved to be the most effective insulin secretagogues among the nine compounds tested at glucose concentrations of 4 and 10 mmol/L [149]. These findings suggest that the number of hydroxyl groups in the anthocyanin ring B significantly influences the ability of β-cells to secrete insulin [150]. Sun *et al*. showed that pre-treating β-cells with bayberry fruit extract rich in C3G (containing 0.5 μmol/L C3G) prevented cell death, enhanced cellular viability, and reduced mitochondrial reactive oxygen species production along with H_2_O_2_-induced cell necrosis [149]. The extracts exhibited a dose-dependent increase in the pancreatic duodenal homeobox 1 gene expression, leading to elevated insulin-like growth factor II gene transcripts and insulin protein expression in pancreatic INS-1 cells.

#### 4.4.4 Improving Lipid Metabolism

Moreover, anthocyanins have been implicated in direct interactions with enzymes integral to lipid metabolism. Studies suggest that anthocyanins may modulate the activity of lipoprotein lipase and hepatic lipogenic enzymes, thereby influencing lipids' synthesis, breakdown, and storage [151]. The lipid-lowering effects of anthocyanins extend to regulating cholesterol levels [152]. Evidence suggests that these compounds may reduce total cholesterol, low-density lipoprotein cholesterol (LDL-C), and triglycerides, collectively contributing to a more favorable lipid profile in individuals with diabetes [153]. Anthocyanins may enhance insulin sensitivity, a critical glucose and lipid metabolism factor [107]. Improved insulin sensitivity can lead to decreased release of free fatty acids and enhanced lipid storage, positively impacting overall lipid balance [154].

Additionally, specific anthocyanins may shape high-density lipoprotein (HDL) function, potentially promoting reverse cholesterol transport. By improving HDL functionality, anthocyanins may contribute to removing excess cholesterol from peripheral tissues, further mitigating dyslipidemia in the diabetic milieu [155]. While these mechanistic insights are promising, ongoing research is essential to elucidate the specific pathways and dose-dependent responses associated with anthocyanin-mediated regulation of lipid metabolism in diabetes mellitus. Nonetheless, these findings underscore the potential therapeutic relevance of anthocyanins as bioactive compounds in managing lipid-related complications in the diabetic context.

Anthocyanins exhibit anti-inflammatory effects by scavenging ROS, modulating NF-κB signaling, and influencing immune response. The antioxidant properties involve scavenging ROS, metal chelation, and Nrf2 pathway modulation. Anthocyanins improve insulin sensitivity, glucose metabolism, and lipid profiles. Additionally, they inhibit carbohydrate-metabolizing enzymes, intestinal glucose transport, DPP-4, and protect β-cells. This multifaceted approach underscores their potential to mitigate diabetic complications.

## 5 EVIDENCE FROM PRECLINICAL STUDIES

In exploring the potential therapeutic impact of anthocyanins in diabetes and its complications, preclinical studies have emerged as pivotal contributors to our understanding of the underlying molecular mechanisms. A wealth of evidence from animal models and *in vitro* experiments has provided valuable insights into the beneficial effects of anthocyanins in mitigating the multifaceted aspects of diabetes. From unraveling the intricate pathways involved in glucose homeostasis to elucidating these natural compounds' anti-inflammatory and antioxidant properties, preclinical studies serve as the foundational framework for establishing the translational potential of anthocyanins in managing diabetes and its associated complications [28, 156, 157].

### 5.1 Animal Models and Experimental Design

Both *in vitro* and *in vivo* studies play pivotal roles in elucidating the intricate mechanisms underlying the impact of anthocyanins on diabetes. *In vitro* investigations meticulously examine cellular responses to anthocyanin exposure, cellular signaling pathways, insulin secretion, and oxidative stress mitigation. These studies provide a foundational understanding of the molecular interactions between anthocyanins and key cellular components relevant to diabetes pathophysiology. Complementing this, *in vivo* studies employing animal models like T2DM mice, streptozotocin-induced diabetic mice/rats, *etc*., serve to bridge the translational gap, offering insights into the systemic effects of anthocyanins on glucose homeostasis, insulin sensitivity, and inflammation [99, 158].

### 5.2 Key Findings on the Influence of Anthocyanins

*In vitro* studies examining the impact of anthocyanins on cultured pancreatic beta cells have demonstrated enhanced insulin secretion and improved glucose uptake. For instance, experiments utilizing INS-1E cells exposed to anthocyanin-rich blueberry extract revealed increased insulin secretion, indicating a potential role in enhancing beta-cell function [159]. Additionally, *in vitro* investigations have explored the molecular mechanisms involved, such as activating AMPK, a key regulator of cellular energy balance [160]. Whereas *in vivo* experiments using diabetic animal models have provided valuable insights. In a study utilizing a streptozotocin-induced diabetic rat model, oral administration of anthocyanins significantly reduced blood glucose levels, improved insulin sensitivity, and prevented oxidative stress [161, 162].

Several *in vitro* studies were carried out to determine the effect of anthocyanins on diabetes. An *in-vitro* study by Barik *et al*. was carried out on Caco-2 cells to determine the effect of anthocyanins from blackcurrant, which inhibited α-glucosidase and modulated glucose uptake and glucose transporters [163]. Another study by Johnson *et al*. [164] concluded that anthocyanins from fermented blueberry-blackberry and blackberry beverages demonstrated the potential to increase insulin secretion from pancreatic β-cells. They also upregulated genes related to GLP-1 and the insulin secretory pathway, along with enhancing protein expression associated with insulin. These findings suggest a promising role for these anthocyanins in T2DM management [164]. Other *in vitro* studies of anthocyanins influencing diabetic parameters have been listed in Table **3**.

Guo *et al*. conducted an *in vivo* study on T2DM mice to study the effect of anthocyanin C3G. They concluded that anthocyanins decreased blood glucose and inflammation levels and increased insulin sensitivity in mice [170]. Other studies indicating the effect of anthocyanins on oxidative stress, inflammation, insulin sensitivity, and lipid modulation are mentioned in Table **4**.

### 5.3 Limitations and Challenges

Apart from ethical concerns, anthocyanins may behave differently in various species due to metabolic and physiological variations. The metabolism of anthocyanins may vary between animals and humans. The pharmacokinetic parameters such as absorption, distribution, metabolism, and excretion may differ [171-184]. The dose considered effective in animals may not directly correlate with a safe and effective dose for humans. A major factor affecting anthocyanins is their stability. They are pH, light, and temperature sensitive and maintaining their stability during the experiments can be challenging. The concomitant drugs might interact with anthocyanins whose data cannot be obtained from animal studies as animal models may not fully represent human physiology, and outcomes observed in animals may only sometimes translate to human clinical benefits.

## 6 CLINICAL STUDIES: TRANSLATING EVIDENCE TO HUMANS

As the promising findings from preclinical studies laid a solid foundation, translating evidence to human subjects through clinical studies becomes crucial in understanding the practical implications of anthocyanins in diabetes management. Clinical studies bridge the gap between laboratory research and real-world applications, providing valuable insights into the safety, efficacy, and potential challenges of incorporating anthocyanins into the therapeutic landscape for diabetes and its complications. These investigations delve into the complexities of human physiology, addressing questions about dosage, bioavailability, and long-term effects. By examining the impact of anthocyanin supplementation on various clinical parameters, including glycaemic control, inflammation markers, insulin sensitivity, and lipid profiles, these studies offer valuable data for healthcare professionals and policymakers seeking evidence-based strategies for diabetes prevention and treatment.

### 6.1 Human Trials on Anthocyanins and Diabetes

The effectiveness of anthocyanins from food sources in controlling plasma glucose, reducing oxidative stress, and increasing insulin sensitivity in diabetic patients has been detailed in several clinical trials [185-188]. In a randomized controlled, four-arm, crossover trial (NCT01199848), 21 adult subjects with insulin resistance were recruited and were randomized to receive beverages containing different doses (*i.e.*, 0, 10, 20, and 40 g) of strawberry powder. Blood was collected at certain time intervals. The study's primary outcome measure was post-prandial insulin levels, and the secondary outcome measure was strawberry’s dose-response effect on post-prandial oxidative stress. It was observed that the post-meal insulin levels significantly reduced after 40 g of strawberry powder ingestion; however, the post-meal glucose concentrations were similar for all the beverages. The study concluded that anthocyanins (pelargonidin) in strawberries reduced the post-prandial insulin demand to manage post-prandial glucose in obese individuals with insulin resistance [189].

Yang *et al*. conducted a clinical trial (NCT02689765) to determine the effect of insulin resistance from Bilberry and blackcurrant on serum adipsin and visfatin in patients with prediabetes or newly diagnosed diabetes. One hundred sixty participants were randomized to receive 320 g of anthocyanins or placebo daily for 12 weeks. The study's primary endpoint was a fasting glucose and glycated haemoglobin (HbA1c) change. The secondary endpoint was the area under the curve for glucose, insulin, and C-peptide releasing test, lipids, calculated pancreatic β-cells function, and insulin resistance. The study showed that purified anthocyanin supplementations increased serum adipsin, decreased serum visfatin, and improved HbA1c compared to the placebo group in prediabetic patients or patients with newly diagnosed diabetes [188].

Another trial (NCT02317211) elaborating the effect of anthocyanins on oxidative stress and insulin resistance in diabetic patients was conducted. Fifty-eight patients were randomized to receive either 320 g of anthocyanins or placebo daily for 24 weeks. The primary and secondary endpoints were glycemic control and biomarkers for oxidative stress like SOD and glutathione peroxidase (GSH-PX), respectively. Observations showed that anthocyanins improved dyslipidemia, enhanced antioxidant capacity, and prevented insulin resistance in subjects with T2DM [189,190]. Other clinical trials depicting the effect of anthocyanins on different parameters of diabetes are mentioned in Table **5**.

Thus, several randomized controlled trials and cross-over clinical trials have been conducted to determine the effect of anthocyanins on diabetes mellitus. The findings from these studies concluded that anthocyanins from sources like blackberry, strawberry, and blackcurrant effectively reduced and prevented DM and its associated complications. The findings include but are not limited to, increased insulin sensitivity, improved HbA1c, enhanced antioxidant and inflammatory capacity, decrease in fasting and post-prandial glucose, and improved lipid profile.

## 7 EFFECTS OF ANTHOCYANINS ON SPECIFIC DIABETES-ASSOCIATED COMPLICATIONS

Delving into the nuanced landscape of diabetes-associated complications, exploring anthocyanins' effects on specific facets of this multifaceted condition unveils a promising avenue for targeted therapeutic interventions. Beyond the overarching impact on glycaemic control and systemic inflammation, anthocyanins exhibit a spectrum of effects on distinct diabetes-associated complications. From retinopathy and neuropathy to nephropathy and cardiovascular complications, a growing body of evidence from preclinical and clinical studies illuminates the potential of anthocyanins to mitigate the intricate mechanisms underlying these specific manifestations. Understanding the nuanced effects of anthocyanins on individual diabetic complications provides a comprehensive perspective, offering insights that could pave the way for tailored interventions in the holistic management of diabetes-related health challenges [36, 191, 192].

### 7.1 Cardiovascular Complications

Cardiovascular complications stand as the primary cause of mortality and disability among individuals with DM [193]. Dyslipidemia, marked by low HDL-cholesterol and elevated LDL-cholesterol and triglycerides concentrations, constitutes a key risk factor for cardiovascular disease in T2DM. DM triggers chronic heart issues, including inflammation, hypertrophy, apoptosis, and fibrosis, leading to compromised cardiac function and potential heart failure (Chen *et al*., 2016). Platelets are key in atherosclerosis and cardiovascular disease (CVD) in DM [192].

Elevated glucose levels contribute to inflammation and myocardial fibrosis, major factors in diabetic cardiac complications. The pro-inflammatory cytokine interleukin-17 (IL-17) is crucial in cardiac fibrosis. Anthocyanins exhibit protective effects by reducing IL-17 expression by regulating miR-214-3p, a non-coding RNA controlling inflammation processes, thereby safeguarding cardiac function. Metalloproteinases (MMPs) promote fibroblast transformation into myofibroblasts, leading to myocardial remodeling. Anthocyanins maintain homeostasis between MMPs and their tissue inhibitors (TIMPs), inhibiting transforming growth factor β (TGF-β) and fibroblast Growth Factor 2 (FGF2) associated with cardiac fibrosis. Additionally, anthocyanins reduce toll-like receptor-4 (TLR4)/NF-κB activation, improving myocardial inflammation and function. In hyperglycemia-induced cardiomyocyte necrosis and apoptosis, anthocyanins enhance IGFI-R/PI3K/AKT survival signaling and B-cell lymphoma 2 (Bcl-2) family-associated anti-apoptotic mechanisms while suppressing caspase family, Bad, and Bak proteins related to apoptosis mechanisms [194-196].

### 7.2 Nephropathy

Diabetic nephropathy (DN) is a major complication of DM and a leading cause of end-stage renal disease globally [197]. The disease progression involves hyperglycemia and inflammation pathways, accumulating extracellular matrix proteins and declining irreversible renal function [198]. Studies have highlighted the potential of natural extracts in mitigating DN. Purple corn extract exhibited significant inhibitory effects on various markers of DN in db/db mice, including glomerular monocyte activation, macrophage infiltration, mesangial hyperplasia, collagen fiber accumulation, and severe albuminuria [199]. C3G treatment in db/db mice showed positive effects, including improved renal function and histology, reduced renal hypertrophy, downregulated glucose levels, and hypolipidemic, antioxidant, and anti-inflammatory effects [200]. Other extracts, such as red-orange, lemon extract, and DIAVIT (blueberry and sea buckthorn extract), demonstrated protective effects against kidney damage and the development of albuminuria [201, 202]. Not all interventions showed consistent results; a different animal experiment did not significantly improve kidney inflammation. *In vitro* studies suggested that protocatechuic acid, an anthocyanin metabolite, may prevent extracellular matrix accumulation and subsequent DN development [172].

Anthocyanins improve renal function by reducing inflammation and enhancing antioxidative capacity [173, 200, 203, 204]. They mitigate NF-κB-mediated renal fibrosis by alleviating oxidative stress [173] and inhibit the Ras/PI3K/Akt pathway, reducing renal mesangial fibrosis [205]. Anthocyanins also block the IL-8/Tyk2/STAT pathway, which promotes renal mesangial fibrosis and inflammatory cell infiltration [199] and inhibits the TGF-β1/Smad2/3 pathway, improving renal fibrosis [204]. Additionally, anthocyanins attenuate kidney cell apoptosis by modulating apoptosis-related proteins and reducing phosphorylation of p38 MAPK and ERK1/2 [201, 206].

### 7.3 Neuropathy

Diabetic neuropathy is a debilitating complication arising from prolonged hyperglycemia in individuals with DM [207]. It manifests as nerve damage, leading to pain, numbness, and impaired motor function, primarily affecting the peripheral nervous system [203, 208]. The etiology of diabetic neuropathy is multifactorial, involving complex mechanisms such as oxidative stress, inflammation, and metabolic abnormalities [209]. Anthocyanins have garnered attention for their potential therapeutic effects in mitigating diabetic neuropathy. The pathogenesis of diabetic neuropathy involves the generation of ROS and inflammation, contributing to nerve damage [210].

Through their antioxidant capacity, Anthocyanins counteract ROS production, thereby attenuating oxidative stress-induced damage to nerve tissues. Furthermore, anthocyanins exhibit anti-inflammatory effects by modulating inflammatory signaling pathways [211]. Chronic inflammation is implicated in the progression of diabetic neuropathy, and anthocyanins have been shown to inhibit proinflammatory cytokines and reduce the activation of inflammatory mediators [212]. Studies have demonstrated that anthocyanin-rich extracts from berries, grapes, and other sources can ameliorate neuropathic symptoms in experimental models of diabetes. These effects may be attributed to the ability of anthocyanins to enhance nerve function, promote nerve regeneration, and alleviate pain associated with diabetic neuropathy [29]. Additionally, anthocyanins may modulate glucose metabolism and improve insulin sensitivity, addressing underlying factors contributing to neuropathic complications in diabetes.

### 7.4 Retinopathy

Diabetic retinopathy, a major complication of diabetes, leads to global vision loss. It has two phases: non-proliferative retinopathy and proliferative retinopathy, characterized by malfunctioning retinal blood vessels and a compromised blood-retinal barrier. These issues result from oxidative stress and chronic inflammation in individuals with diabetes. Studies using streptozotocin-induced rats and diabetic mice showed promising results in mitigating retinal complications. Song *et al*. studied streptozotocin-induced rats, showing that blueberry anthocyanins improved blood-retinal barrier integrity in rats [213]. Bilberry extracts reduced fluorescein leakage and lowered rats' vascular endothelial growth factor (VEGF) levels [214]. Moreover, C3G in mice attenuated retinal vascular leakage and inhibited glial cell proliferation and angiogenesis [215]. These findings highlight the potential of anthocyanins, bilberry extract, and C3G in addressing diabetic retinopathy.

Anthocyanins exhibit potential protective effects on the retina, which can be attributed to their antioxidant and anti-inflammatory properties. Specifically, these compounds may modulate the Nrf2/heme oxygenase-1 signaling pathway, enhancing antioxidant capacity and mitigating inflammation in retinal cells [213]. Moreover, anthocyanins have demonstrated the ability to suppress both the mRNA and protein expression of VEGF, a factor associated with blood-retinal barrier breakdown, thereby preventing the onset of diabetic retinopathy [214]. Microglia, resident monocytes in the retina, are pivotal in diabetic retinopathy [216]. Anthocyanins have been found to regulate the activation of microglia, contributing to the delay in the progression of diabetic retinopathy. Additionally, these compounds enhance retinal vascular permeability by upregulating the expression of tight junction proteins, including occludin-1, claudin-1, and ZO-1 [215].

## 8 INTERACTION AND SYNERGISTIC EFFECTS WITH CONVENTIONAL THERAPIES

The interaction between anthocyanins and anti-diabetic medications is a crucial aspect to consider when exploring the potential benefits of anthocyanins in managing diabetes and its complications. While anthocyanins hold promise in supporting overall health, understanding their interaction with anti-diabetic medications is essential for ensuring safe and effective diabetes management. One of the primary concerns when examining the interaction between anthocyanins and anti-diabetic medications is their potential to affect blood glucose levels.

The synergistic effect of anthocyanins and anti-diabetic medications could lead to better blood glucose control, reducing the reliance on high doses of anti-diabetic drugs and minimizing potential side effects. Additionally, anthocyanins' anti-inflammatory properties may contribute to alleviating the chronic inflammation often associated with diabetes, further supporting the overall management of the condition. However, it is crucial to note that the interaction between anthocyanins and anti-diabetic medications may not always be straightforward [217, 218]. This underscores the importance of individualized treatment plans and close monitoring of blood glucose levels for individuals concurrently consuming anthocyanin-rich foods or supplements alongside anti-diabetic medications.

Moreover, the metabolism of both anthocyanins and anti-diabetic medications occurs in the liver, raising the possibility of interactions at the metabolic level [217, 218]. The potential for competition for the same metabolic pathways or enzymes could influence the bioavailability and efficacy of either anthocyanins or anti-diabetic drugs. Further research is needed to elucidate these metabolic interactions and guide healthcare professionals in optimizing treatment strategies. Individuals with diabetes should consult their healthcare providers before incorporating anthocyanin-rich foods or supplements into their regimen, especially if they are taking anti-diabetic medications. Healthcare professionals can assess potential interactions based on the specific medications prescribed, considering factors such as dosage, frequency, and the individual's overall health status. The interaction between anthocyanins and anti-diabetic medications is complex and multifaceted and requires careful consideration. While anthocyanins hold promise in complementing diabetes management, potential interactions and their impact on blood glucose levels must be thoroughly examined. A collaborative approach between healthcare providers and individuals with diabetes is crucial to ensure a well-informed and personalized treatment plan that maximizes the benefits of anthocyanins and anti-diabetic medications while minimizing potential risks.

## 9 SAFETY CONSIDERATIONS

Ensuring the safety of anthocyanins is paramount, especially considering their increasing popularity as potential health-promoting compounds. While research has highlighted numerous potential benefits [34, 219], addressing safety considerations associated with anthocyanin consumption is essential to guide individuals, healthcare professionals, and regulatory bodies. One primary concern lies in the potential for allergic reactions, albeit rare, in individuals with known allergies to specific anthocyanin-rich foods. Careful monitoring and immediate medical attention in case of adverse reactions are imperative. Some Chinese herbal medicines may interact with certain medications, particularly anticoagulants and antiplatelet drugs, increasing the risk of bleeding [220]. Individuals on blood-thinning medications should consult healthcare providers before introducing anthocyanin-rich foods or supplements. The metabolic effects of anthocyanins on blood glucose levels, especially in diabetic individuals, should be monitored closely to avoid potential hypoglycemia. Gastrointestinal distress may occur with excessive anthocyanin consumption, making moderation advisable, particularly for those prone to digestive issues. The purity and quality of anthocyanin supplements are vital to prevent potential health risks associated with contaminants or undisclosed ingredients. Individual variability in responses to anthocyanins, influenced by age, health status, and genetics, underscores the importance of monitoring and adjusting intake based on individual needs.

Additionally, potential food sensitivities to anthocyanin-rich foods should be considered, and individuals with known sensitivities should exercise caution. Some anthocyanin-rich foods, such as berries, may also contain oxalates, potentially contributing to kidney stone formation in susceptible individuals [221]. Lastly, maintaining open communication with healthcare professionals and adopting a balanced and varied diet can help individuals harness the potential benefits of anthocyanins while minimizing possible risks. However, anthocyanin exhibits a broad spectrum of safety attributes as a food additive. Moreover, empirical data indicates that anthocyanins in consumed food items constitute intricate flavonoid combinations, possessing characteristics of non-toxicity and non-mutagenicity [35].

Furthermore, these bioflavonoids manifest diverse effects that are conducive to maintaining human health. Several clinical trials have investigated the safety of anthocyanins, particularly in the context of diabetes. The findings suggest that anthocyanins are generally safe for consumption in individuals with diabetes [222].

## 10 CHALLENGES AND FUTURE DIRECTIONS

Navigating the promising landscape of anthocyanins in the context of diabetes and its complications presents several challenges that warrant attention. One major obstacle involves the need for more comprehensive clinical trials and longitudinal studies to establish a clearer cause-and-effect relationship between anthocyanin consumption and diabetes management. Existing research often involves varying anthocyanin dosages, making it challenging to determine optimal intake levels for consistent health benefits [185-187]. Furthermore, addressing the individual variability in responses to anthocyanins and their potential interactions with specific medications requires a nuanced approach to treatment strategies.

Theoretical support is provided for integrating berry fruits, cherries, and purple sweet potatoes into high-value functional foods like anthocyanin-rich rice [223]. However, the lack of a systematic exploration of the structure-activity relationship between anthocyanins and their targets is evident. The mechanisms by which anthocyanins reduce intestinal glucose transport remain unclear, requiring further identification of DPP-4 inhibitors among specific anthocyanins. Confirmation of the impact of acylated anthocyanins from purple sweet potatoes on β-cell protection and insulin secretion demands more evidence [224]. Investigating whether different anthocyanin structures affect β-cells through diverse mechanisms or intracellular signaling pathways is crucial. Although anthocyanins may aid in hyperglycemia control by regulating glucose homeostasis, uricemia, glucose transport, insulin secretion [225], and gut microbiota [226], comprehensive research on their effects, mechanisms, and microbial metabolites is necessary.

Further exploration is needed to understand the potential of anthocyanins in regulating the combination of hyperuricemia and hyperglycemia. These strategies enhance our understanding of anthocyanins' efficacy and biological properties. Given a case report linking black cherry anthocyanins to acute kidney injury in a patient with chronic kidney disease secondary to T2DM [227], dietary guidelines for anthocyanins are crucial. Therefore, additional clinical trials are imperative to evaluate the recommended consumption of anthocyanins in individuals with diverse health conditions.

As we navigate the challenges associated with anthocyanins in the context of diabetes, several promising avenues beckon for future exploration. Investigating the synergistic effects of anthocyanins with other bioactive compounds in fruits and vegetables could unveil more comprehensive strategies for diabetes management [228]. Moreover, focusing on personalized nutrition approaches considering individual variations in response to anthocyanins can pave the way for tailored dietary recommendations. Harnessing advanced technologies, such as metabolomics and nutrigenomics, holds immense potential in unraveling the intricate mechanisms underlying anthocyanin metabolism and their impact on diabetes-related pathways [229]. Collaborative efforts between researchers, healthcare professionals, and regulatory bodies are pivotal for establishing evidence-based guidelines that can unlock the full potential of anthocyanins in preventing and managing diabetes and its complications.

The prospects for anthocyanin-based therapies in DM are promising, propelled by an expanding body of scientific evidence elucidating their multifaceted effects. Ongoing research endeavors aim to unravel the intricate molecular mechanisms by which anthocyanins influence glucose homeostasis, enhance insulin sensitivity, and safeguard pancreatic beta-cells, paving the way for targeted therapeutic interventions. Additionally, the modulation of the gut microbiota by anthocyanins presents a novel area of exploration with potential implications for metabolic outcomes. As the field advances, a personalized medicine approach, considering individual variability in response, and optimization of anthocyanin formulations for improved bioavailability are emerging as key considerations. Furthermore, investigations into synergies with existing anti-diabetic medications and rigorous clinical trials will be pivotal in substantiating the safety and efficacy of anthocyanin-based therapies across diverse patient populations. The future trajectory involves a comprehensive understanding of anthocyanins' molecular actions, innovative formulation strategies, and robust clinical validation to realize their therapeutic potential in diabetes management.

## CONCLUSION

The comprehensive exploration of the potential therapeutic role of anthocyanins in the context of DM underscores their multifaceted impact on the disease and its associated complications. Anthocyanins exhibit promising effects on glucose homeostasis, insulin sensitivity, and oxidative stress, presenting a potential avenue for preventing and managing diabetes. The diverse isoforms of anthocyanins, such as cyanidin, delphinidin, pelargonidin, peonidin, malvidin, and petunidin, contribute to the extensive variability in their biological activities. Importantly, clinical trials have demonstrated the efficacy of anthocyanins in controlling plasma glucose, reducing oxidative stress, and enhancing insulin sensitivity in diabetic patients, thus validating their potential therapeutic benefits. Moreover, anthocyanins showcase safety as food additives, providing reassurance for their utilization in individuals with diabetes. The intricate molecular mechanisms elucidated in several studies highlight the versatility of anthocyanins in targeting key aspects of diabetes pathophysiology, ranging from carbohydrate metabolism and insulin resistance to inflammation and oxidative stress. This wealth of evidence positions anthocyanins, abundant in colored fruits, vegetables, and other natural sources, as promising bioactive compounds for developing complementary strategies in the holistic management of diabetes and its associated complications. As we unravel the complexities of this metabolic disorder, anthocyanins emerge as a beacon of hope, offering a natural and safe approach to optimizing glucose regulation and facilitating the diverse complications associated with diabetes.

In closing remarks, the comprehensive exploration of anthocyanins in this review reveals their multifaceted impact on diabetes and associated complications. From foundational preclinical insights to compelling *in-vivo* studies and insightful clinical investigations, anthocyanins emerge as promising therapeutic agents. Their ability to modulate glucose homeostasis, attenuate oxidative stress, and curb inflammation positions them as valuable contributors to diabetes care. As we navigate the landscape of personalized medicine, ongoing research holds the key to fully harnessing the potential of anthocyanins, shaping a nuanced and effective approach to preventing and managing diabetes-related complications.

## Figures and Tables

**Fig. (2):**
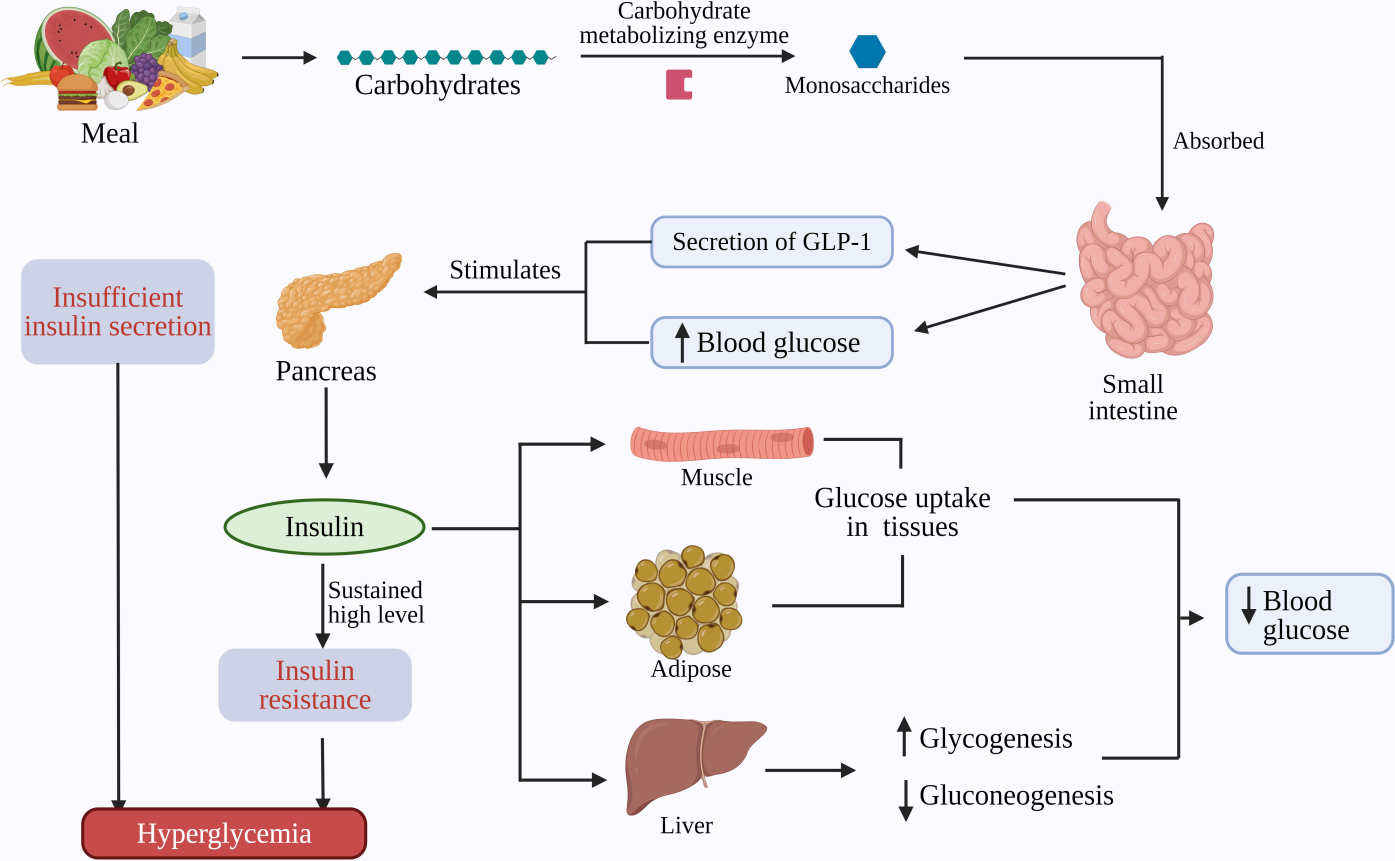
Regulation of glucose homeostasis (Created in BioRender.com).

**Fig. (3):**
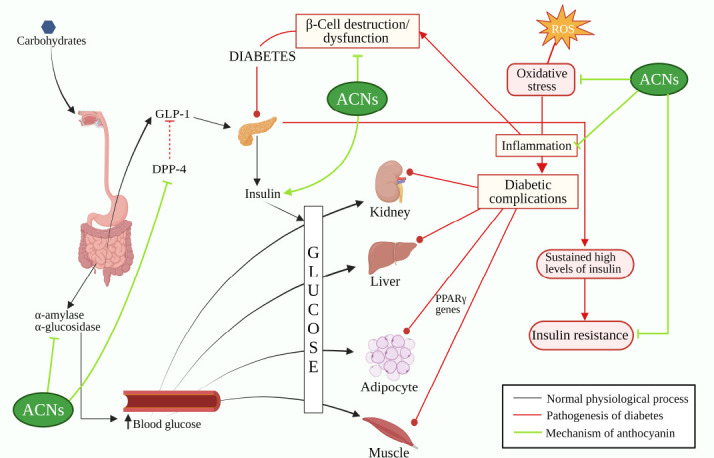
Pathogenesis of diabetes and mechanism of anthocyanins in ameliorating diabetes (Created in BioRender.com).

**Table 1:** Anthocyanidin isoforms and associated pigment colors.

**Isoform**	**Pigment**
Cyanidin	Reddish-purple (magenta)
Delphinidin	Blue-reddish or purple
Pelargonidin	Red-orange
Peonidin	Magenta
Malvidin	Purple
Petunidin	Dark red or purple

**Table 2:** Anthocyanin composition in various fruits.

**Source**	**Anthocyanin**
Apple	Cy 3-galactoside, Cy 3G, Cy 3A, Cy 3-xyloside, acylated derivatives
Elderberry	Cy 3-sambubioside, Cy 3G, Cy 3-sambubioside-5-glucoside, Cy 3-diglycoside
Blackberry	Cy 3G, Cy 3-rutinoside
Blackcurrant	Cy 3G and Dp 3-glucosides and 3-rutinosides, Cy 3-sambubioside
Cherry	Cy 3G, Cy 3-rutinoside
Cranberry	Cy 3-galactoside, Cy 3G, Cy 3A, Pn 3-arabinoside, Pn 3-galactoside
Grapes	Mv 3-glucoside, Dp 3-glucoside, Pt 3-glucoside, Pn 3-glucoside, 3-glucoside, Mv 3-glucoside-acetate
Plum	Cy and Pg 3-rutinosides, Cy 3G
Raspberry	Cy 3G, Pg 3-glucosides, Cy 3-rutinosides, Pg 3-rutinosides, Cy 3-sophorosides, Pg 3-sophorosides, Cy 3-glucorutinosides and Pg 3-glucorutinosides
Redcurrant	Cy 3G, Cy 3-rutinosides and others
Blueberry	Mv 3-galactoside, Dp 3-galactoside, Dp 3-arabinoside, Pt 3-galactoside, Pt 3-arabinoside, Mv 3-arabinoside
Strawberry	Pg 3-glucosides and Cy 3G
Pear	Cy 3G, Cy 3A

**Abbreviations:** Cy: Cyanidin; Dp: Delphinidin; Pg: Pelargonidin; Pt: Petunidin; Mv: Malvidin; Pn: Peonidin; Cy 3G: Cy 3-glucoside; Cy 3A: Cy 3-arabinoside.

**Table 3:** The anti-diabetic properties of anthocyanins: Results of representative *in vitro* studies.

**Source**	**Anthocyanins**	**Model**	**Results**	**References**
Anthocyanin-rich extract from blackcurrant	Dp, Cy, Pt, Cy 3G, Dp 3G.	Caco-2 cells	Inhibited α-glucosidase, ↓ plasma glucose, insulin, GIP, and GLP-1.	[98, 163]
Anthocyanin extracts from fermented berry beverages	Dp 3A, Mv 3A, Cy 3A, Pt 3G	INS-1E beta cells and Caco-2 cells	↑ Glucose-stimulated insulin secretion, Up-regulated GLP-1, and insulin secretory pathway genes.	[164]
Purple sweet potato extract	Peonidin 3-(60,600-dicaffeoyl sophoroside)-5-glucoside, Peonidin 3-(60-caffeoyl-600-hydroxybenzoic sophoroside)-5- glucoside, Peonidin 3-(60-caffeoyl-600-feruloyl sophoroside)-5-glucoside.	Huh7 and HepG2 cells	Inhibition of α-amylase, α -glucosidase. Induced the Nrf2, enhanced cellular glutathione concentrations. ↓ Lipid peroxidation in cultured hepatocytes.	[165]
Individual compounds from mulberry	Cy 3G	MIN6N beta cells	Protected from glucotoxicity or apoptosis. Restored the cell viability. Suppressed the MAPKs activation and regulated the intrinsic apoptotic pathway-associated proteins. Improved insulin secretion under high glucose or H_2_O_2_ conditions.	[166]
Individual compounds	Cy	INS-1E beta cells	Stimulated insulin secretion. ↑ Intracellular Ca^2+^ signals. Activated the VDCCs. ↑ Expression of GLUT2, Kir6.2, and Cav1.2.	[167]
Mulberry anthocyanin extract	Cy 3G, Cyanidin 3-rutinoside, Pelargonidin 3-glucoside.	HepG2 cells	↑ Glucose uptake and consumption, and glycogen content. ↓ Gluconeogenesis, ↓ Phosphoenolpyruvate carboxykinase and glucose-6-phosphatase activities. ↑ Phosphorylation of AKT and glycogen synthase kinase-3β, glycogen synthase 2 expression.	[168]
Lowbush blueberry fruits	Fermented juice	Insulin-sensitive cultured muscle cells and adipocytes	↑Glucose uptake and insulin sensitivity.	[169]

**Abbreviations:** Dp: Delphinidin; Cy: Cyanidin; Pt: Petunidin; Mv: Malvidin; Cy 3G: Cy 3-glucosides; Dp 3G: Dp 3-glucoside; Dp 3A: Dp 3-arabinoside; Mv 3A: Mv 3-arabinoside; Cy 3A: Cy 3-arabinoside; Pt3G: Pt 3- glucosides; GIP: Gastric inhibitory polypeptide; GLP‐1: Glucagon‐like peptide‐1; Nrf2: Nuclear factor erythroid 2-related factor 2; ROS: Reactive oxygen species; MAPKs: Mitogen-activated protein kinases; VGCC: Voltage-gated calcium channels; GLUT2: Glucose transporter 2; Kir 6.2: A subunit of the ATP-sensitive K+ channel; Cav1.2: Alpha-1 subunit of a voltage-dependent calcium channel; AKT: Serine/threonine protein kinase.

**Table 4:** The anti-diabetic properties of anthocyanins: Results of representative *in vivo* studies.

**Source/ Anthocyanins**	**Dose**	**Model**	**Results**	**References**
Cy 3G	0.2% of the diet	High-fat diet-fed C57BL/6J mice	↓ Blood glucose, ↑ Insulin sensitivity, ↓ TC and steatosis in the liver, ↓ Inflammation levels	[170]
Bilberry extract	10 g/kg of diet	Male KK-A*^y^* mice	↓ Blood glucose, ↑ Insulin sensitivity, ↓ Glucose production and lipid content in the liver	[171]
Black soybean seed coat extract (BSSCE)	100, 200, 400 mg of BSSCE/kg of body weight /day	Streptozotocin-induced T2DM mice model	↓ Fasting blood glucose levels, ↓ Insulin levels and HOMA-IR, ↑ HOMA-β, ↑ Glycogen content in the liver and muscle, ↓ NEFA, TG, and TC. ↑ HDL-C level, ↓ Liver, kidney, and pancreas damage.	[172]
*Myrciaria cauliflora* extracts	0.1, 0.5 and 1% in diet	Streptozotocin-induced diabetic mice model	↓ Oxidant and inflammatory damage in the kidney and renal fibrosis	[173]
Purple sweet potato	200, 500 mg/kg of body weight per day	Streptozotocin-induced T2DM mice model	↓ Blood glucose, ↑ Glucose tolerance, ↓ LDL-C, TG, and TC, ↑ HDL-C level, ↓ Liver damage, ↑ Antioxidant capacity	[174]
Cy 3G	150 mg/kg	Diabetic db/db mice	↓ Fasting blood glucose levels and body weight gain, ↓ Pancreas damage, ↓ ROS level	[175]
Purple sweet potato extracts	-	High-fat diet-fed mice	Improved the fasting blood glucose level, glucose, and insulin tolerance. ↓ ROS production. Restored GSH content and antioxidant enzyme activities. ↓ ER stress in the livers. Suppressed the c-Jun-N-terminal kinase 1 and kappa B kinase b activation and NFκB/ p65 nuclear translocation. Restored the impairment of the insulin receptor substrate 1/ phosphoinositide 3 kinase/Akt insulin signaling in the livers.	[176]
Aronia Berry extract (ABE)	10 or 100 mg of ABE/ Kg of body weight	Low-dose streptozotocin-induced T1DM mice model	↑ Insulin secretion maintains the round shape of the pancreas, protecting pancreatic beta cells. ↓ Sucrase & maltase activity, ↓ LDL-C and TG.	[177]
Blueberries powder	4% BB in diet	High-fat diet-fed Male C57BL/6 mice	↓ Insulin resistance, hyperglycemia	[178]
Cyanidin 3-O-beta-D-glucoside	2 g of Cyanidin 3-O-beta-D-glucoside/ Kg of diet	Hepatic ischemia-reperfusion in rats	Protective effect against oxidative stress	[179]
Purple sweet potato (*Ipomoea batatas* cultivar Ayamurasaki)	100 mg/kg of body weight	Sprague-Dawley rats	↓ α-glucosidase activity ↓ Plasma insulin	[180]
*Vaccinium arctostaphylos* fruit extract (VFE)	200 or 400 mg VFE/ Kg of body weight	Alloxan-diabetic male Wistar rats.	↓ Plasma glucose, ↑ Insulin and GLUT4 expression, ↓ α-glucosidase activity, ↑ Antioxidant enzymes activities (SOD, CAT and GPx)	[181]

**Abbreviations:** Cy 3G: Cyanidin-3-glucoside; T2DM: Type 2 diabetes mellitus; HOMA-IR: Homeostatic Model Assessment for Insulin Resistance; HOMA-β: Homeostasis model assessment of β-cell function; NEFA: Non-esterified fatty acid; TC: Total cholesterol; TG: Triglyceride; HDL-C: High-density lipoprotein cholesterol; LDL-C: Low-density lipoprotein cholesterol; ROS: Reactive oxygen species; GSH: Glutathione; ER: Endoplasmic reticulum; GLUT4: Glucose transporter 4; SOD: Superoxide dismutase; CAT: Catalase; GPx: Glutathione peroxidase.

**Table 5:** Completed and ongoing clinical trials on anthocyanins and their effect on diabetes mellitus.

**Trial No.**	**Subjects (*n*)**	**Type of Patients**	**Trial Status**	**Anthocyanin Source**	**Dose**	**Effects**
NCT01199848	21	Adults with IR, high fasting blood glucose	Completed	Strawberry	10, 20, and 40 g	↑ Insulin sensitivity
NCT02689765	160	T2DM, IR, glucose metabolism disorder, lipid metabolism disorder	Completed	Bilberries, Blackcurrants (ACN capsules)	320 g/day	↑ Serum adipsin, ↓ Serum visfatin, Improved HbA1C and apolipoprotein A1 and B.
NCT02317211	58	T2DM	Completed	Purified ACNs	320 mg/day	↑ Antioxidant capacity, ↓ Fasting plasma glucose, serum LDL-C, and apolipoprotein B-48.
NCT01180712	16	Male, T2DM, controlling diabetes by di*et al*one	Completed	Blackberry extract	1.4 g/day	↓ Post-prandial glycaemic response and OGTT. ↑ Insulin sensitivity.
NCT05399134	20	T2DM, HbA1C <10%, not on prandial insulin therapy	Completed	ACN fortified bred	-	Primary outcome: Post-prandial glycaemic response
NCT01245270	08	T2DM Male	Completed	Bilberry	0.47 g/day	↓ Postprandial glycemia and insulin
NCT01766570	60	T2DM, IR	Completed	Strawberry and cranberry extract	1.84 g/day	Primary outcomes: change in cardiometabolic state
NCT02291250	16	Obese male or female	Ongoing	Blackcurrants	200 g/day	Primary outcomes: Plasma glucose area under the curve Secondary outcomes: Plasma insulin area under the curve
NCT01564498	60	Male/Female abnormal fasting glucose	Ongoing	Purple potato, orange carrot, Purple carrots	300 – 500 mg 200 – 300 mg 200 – 300 mg	Secondary outcome: Insulin resistance
NCT05956106	40	Fasting blood glucose levels >10 mg/dl	Ongoing	Strawberry and cranberry extract	45 g/day	Primary outcomes: fasting blood glucose, Lipid profile

**Abbreviations:** IR: Insulin resistance; T2DM: Type 2 diabetes mellitus; ACN: Anthocyanins; HbA1C: Glycated haemoglobin; LDL-C: Low-density lipoprotein-cholesterol; OGTT: Oral glucose tolerance tests.

## LIST OF ABBREVIATIONS

AGEs: Advanced Glycation End-Products
DM: Diabetes Mellitus
SOD: Superoxide Dismutases
T1DM: Type 1 Diabetes

## TRANSPARENT REPORTING ON AI AND AI-ASSISTED TECHNOLOGIES

We have used Grammerly.com (Premium version) for grammatical checks and language improvement.
